# Elderly-onset rheumatoid arthritis vs. polymyalgia rheumatica: Differences in pathogenesis

**DOI:** 10.3389/fmed.2022.1083879

**Published:** 2023-01-12

**Authors:** Jinzhi Wu, Fan Yang, Xinlei Ma, Jin Lin, Weiqian Chen

**Affiliations:** Division of Rheumatology, The First Affiliated Hospital, Zhejiang University School of Medicine, Hangzhou, Zhejiang, China

**Keywords:** elderly-onset rheumatoid arthritis, polymyalgia rheumatica, rheumatoid arthritis, pathogenesis, inflammation

## Abstract

Rheumatoid arthritis is a chronic autoimmune disease that mainly affects the facet joints. Elderly-onset rheumatoid arthritis appears to exhibit symptoms similar to those of polymyalgia rheumatica, characterized by morning stiffness and pain in the shoulder and hip joints. Both diseases develop in the elderly, and it is sometimes challenging to distinguish them. Here, we identify the differences in pathogenesis between elderly-onset rheumatoid arthritis and polymyalgia rheumatica to assist with a clear differential diagnosis and effective early intervention.

## Introduction

Rheumatoid arthritis (RA) is a chronic inflammatory disease that affects synovial tissue and can lead to ongoing joint damage and irreversible disability (1). RA is most likely to affect the hands, feet, and knees. The most common clinical features of RA are pain, joint swelling, and stiffness. Rheumatoid factor (RF) and anti-citrullinated protein antibodies (ACPA) are two crucial antibodies in patients with RA and are used for diagnosis of the condition (2). Elderly-onset rheumatoid arthritis (EORA) is defined as RA with onset occurring when the patient is over 60 (3–5) or 65 (6) years of age, representing 10–33% (4) of cases of RA. Compared with early-onset rheumatoid arthritis, EORA is more evenly distributed across genders, and is associated with a more acute abrupt onset, larger proximal joint involvement (such as the knee and shoulder), higher erythrocyte sedimentation rate (ESR), higher likelihood of weight loss, lower RF positivity, and poorer disease outcomes (3–5, 7). Moderate to severe proliferative synovitis of the shoulder bursae is more often detected in EORA with PMR-like symptoms than in PMR (8). The pathogenesis of EORA may differ from that of early-onset rheumatoid arthritis. EORA also has a different proinflammatory cytokine profile, including higher IL-6 and lower TNF-α, and the susceptibility gene differs in the case of EORA (5). Finally, the geno–phenotype frequencies vary between EORA and early rheumatoid arthritis.

Since EORA exhibits polymyalgia rheumatica (PMR)-like symptoms, it is sometimes difficult to differentiate it from PMR, which is a chronic inflammatory rheumatic disorder commonly occurring in people over 50 years of age and usually with an acute onset. PMR may occur independently or with giant cell arteritis. The primary manifestations of PMR are musculoskeletal pain and morning stiffness in the shoulders and pelvic girdle. Due to a lack of specific antibodies such as RF and ACPA, diagnosis of PMR is generally based on clinical features and an increase in ESR and C-reactive protein (CRP). PMR responds dramatically to a low dose of glucocorticoids (9–12). Meanwhile, EORA responds well to a relatively high dose of glucocorticoids (GC) in the early disease phase.

As PMR and EORA have similar clinical manifestations and their response to glucocorticoid treatment is comparable, physicians should exercise care in distinguishing the two diseases. In this paper, we present a comprehensive review of the published literature and identify the differences in pathogenesis between EORA and PMR.

## Genetic background

Multiple genes are involved in the pathogenic processes of RA and PMR. Thus far, approximately 100 loci related to the risk of RA have been identified, of which human leukocyte antigen (HLA)-DRB1 is the most important. HLA-DRB1 was believed several decades ago to be associated with the risk of RA, as proposed in the shared epitope (SE) hypothesis (13); this claim has been validated by several studies (14–16). HLA-DRB1 has been found to be linked to the T lymphocyte function of recognizing self-reactive peptides (17). HLA-DRB1 has also been linked to susceptibility to PMR; however, different phenotypic frequencies are involved. It has been reported that HLA-DRB1^*^01 occurs at a higher frequency in patients with EORA, while several studies have indicated that HLA-DRB1^*^04 and HLA-DRB1^*^13/14 are more closely related to PMR (18, 19).

Protein tyrosine phosphatase non-receptor (PTPN22) is another important gene that contributes to the development of RA by downregulating the activity of T cells, by preventing variant phosphatase from combining with recombinant C-Src tyrosine kinase (Csk). PTPN22 occurs at a higher frequency in RF-positive patients than in RF-negative patients (20). Although one study has found that the PTPN22 functional variant R620W may be a genetic susceptibility factor for GCA (21), the relationship between PTPN22 and PMR has not been investigated. Protein-arginine deiminase type-4 (PADI4) is a significant RA susceptibility gene in geriatric Asian individuals (22), but not in European individuals (23). CCR6 and TNFAIP3 have also been linked to RA, according to a genome-wide association study (GWAS) meta-analysis (15).

## Immunosenescence

Immune senescence (also termed immunosenescence) is characterized by the immune dysfunction that occurs with aging. It has three main immunological manifestations: first, the immune response to endogenous and exogenous antigens is impaired; second, the immune protection effect is weakened, e.g., the antitumor effect declines or disappears; and third, the established immune memory response is affected, producing a decrease in or disappearance of the response to vaccination. In the process of immune aging, both the innate and the adaptive immune systems of the body will be affected. However, at present, research shows that aging has a greater impact on the adaptive immune system than on the innate immune system. Additionally, thymic involution is a crucial aspect of immunosenescence. Reduced thymus output and a decline in TCR diversity are the results of structural changes and functional thymus atrophy (24). An expansion of senescent CD4^+^CD28^−^T cells and CD8^+^CD28^−^T cells with shortened telomeres has been observed in RA patients (25–27). Moreover, T cell repertoire shrinks significantly as a result of the amplification of these clones (28). Levels of T cell receptor rearrangement excision circles (TRECs) in CD4^+^T cells, which can be used as a measure of the thymic output of T cells, have been found to be significantly lower in RA patients than in age-matched control donors (29). Immunosenescence may be a factor in the diminished thymus function observed in RA patients. Recently, a novel CD28^−^CD4^+^Foxp3^+^ subset of Treg cells (CD28^−^Treg) has been described in circulation in RA. These CD28^−^Treg cells exhibit a deficiency in control function compared to CD28^+^Treg cells and are associated with aging in both RA patients and healthy individuals (30). It has also been discovered that RA patients of all ages have an elevated rate of telomeric erosion. In addition, patients harboring the HLA-DRB1 gene tend to have shorter telomere lengths, which raises the possibility that HLA-DRB1 is related to accelerated telomere erosion in RA (31). Patients with PMR also exhibit higher levels of senescent T cells that express NKG2D, a hallmark of immunosenescence (32).

## Innate immune response

The innate immune system, which includes neutrophils, dendritic cells, and NK cells as well as macrophages (monocytes), is crucial in the development of RA; in turn, it activates adaptive immunity to play a role in the disease. Although not as clear as in the case of RA, the role of the innate immune system in PMR has also been investigated.

The innate immune system is activated when individuals carrying the RA risk gene are exposed to a risky environment (such as *via* smoking). By stimulating the production of cytokines, chemokines, and degradative enzymes, and causing joint destruction, macrophages play a crucial role in the progression of RA (33). There are two types of macrophages: inflammatory phenotype macrophages (M1) induced by lipopolysaccharide and interferon-γ, and anti-inflammatory macrophages (M2) induced by interleukin-4 (IL-4) or IL-13 (34). Increased levels of TNF-α and IL-1, both of which are typically released by M1 macrophages, have been observed in the synovium in RA (35). Recently, it has also been discovered that IL-1β can drive naïve CD4^+^ T cells to differentiate into osteoclastogenic regulatory T cells (O Tregs) *in vivo*. In contrast to the typical suppressive role played by Tregs, O Tregs can overexpress RANKL and cause bone erosion (36). The phagocytic function of macrophages diminishes in the aging population (37). Cytokine secretion is also significantly delayed, which results in defective responses to PAMPs and DAMPs by macrophages (37). However, no specific study has focused on macrophages or monocytes in EORA; thus, more data are urgently needed in this area. Anti-TNF-α therapy for RA has been found to reduce the formation of matrix metalloproteinase (MMP), activate leukocytes, reduce angiogenesis, and alleviate pain (1). TNF-α is higher in EORA patients than in PMR patients (although it is still higher in the latter group than in healthy controls) (38, 39); however, there is no difference in IL-1 levels between EORA and early-onset rheumatoid arthritis (39). Transforming growth factor-1 (macrophage-derived) and interleukin-1 (IL-1) have been found in the temporal artery in PMR (40). Meanwhile, the function of TNF-α in PMR is not fully understood (41). One study has reported observing no significant difference in TNF-α between PMR patients and HCs (42), while another has indicated that TNF-α is much higher in patients with PMR (38). Through measurement of methylome and transcriptome profiles, it has recently been demonstrated that a more proinflammatory phenotype of circulating CD14^+^ monocytes occurs in patients with GCA; the investigators also found that IL-11, a cytokine that can promote the development of Th17 cells, may activate GCA CD14^+^ monocytes (43). Monocyte levels are higher in patients with PMR (44). It can be assumed that CD14^+^ monocytes may also contribute to the inflammatory reaction in PMR.

Neutrophils are also crucial in the pathophysiology of RA. In the early stage, they migrate into the articular cavity, become activated, and exert their function in an inflammatory milieu. The ability of neutrophils to form neutrophil extracellular traps (NETs) actively triggers the immune response of RA *via* autoantigen production against citrullinated proteins (45). It is well known that the function of phagocyte and cytokine production in neutrophils is downregulated in older people (46). Neutrophils from patients with active EORA have been found to exhibit a higher capacity to migrate, but this is not the case in PMR. Neutrophils and monocytes with phagocytic activity have been found to be similar among PMR and EORA patients, and among age-matched HCs. In contrast, oxidative burst is significantly reduced in neutrophils and monocytes from patients with PMR or EORA; however, more data are essential to confirm this result (47). The number of neutrophils has been found to be higher in PMR than in controls, but this number falls following GC treatment, suggesting that the number of neutrophils may be related to the severity of the disease (44).

Toll-like receptors (TLRs) are involved in the recognition of pathogen-associated molecular patterns and the generation of antibodies. For instance, TLR-2 and TLR-4 play an essential role in the persistence of joint inflammation. In comparison to osteoarthritis, increased expression of TLR-2 and TLR-4 has been observed on the surface of monocyte cells from the synovium of RA patients. Both IL-12 and IL-18 can upregulate the production of TLR-2 and TLR-4 in the presence of IFN-γ. Furthermore, an increase in TLR-2/TLR-4 levels can influence downstream processes, such as by enhancing the production of TNF-α and IL-6, both of which promote bone erosion by inducing the secretion of RANKL (48, 49). However, blocking the TLR-4 pathway alone may not benefit patients with RA (50). TLR-7 has been found to be more abundant in circulating mononuclear cells from patients with PMR compared to healthy controls, but its ability to influence the production of proinflammatory cytokines from monocytes diminishes throughout the course of disease activity and reverses during the remission period (51).

IL-6 is another important proinflammatory cytokine in the pathogenesis of RA. It can cause bone destruction by stimulating osteoclast differentiation and suppressing osteoblast maturation, as well as by inducing Th17 differentiation and blocking Treg production (52). IL-6 has been found to be abundant in the synovial tissue and peripheral blood of RA patients, and also in the peripheral blood of EORA patients (53). Compared to methotrexate (MTX) treatment alone, a combination of MTX and tocilizumab effectively reduces the progression of joint deterioration (54). Patients with PMR have higher levels of circulating IL-6 when compared to patients with EORA (38). In the subacromial–subdeltoid bursa of patients with PMR, IL-6 has been found to be derived from various cells (endothelial cells, fibroblasts, and macrophages) (55). Additionally, serum IL-6 originating from CD14^+^ monocytes is significantly correlated with CRP, ESR levels, and disease activity (19, 56). A prospective longitudinal study has found that tocilizumab treatment significantly reduces polymyalgia rheumatica activity score (PMR-AS) (57), and another study has demonstrated that genetically proxied IL-6 receptor inhibition is associated with a lower risk of PMR (58).

Dendritic cells (DCs) exert their functions through antigen presentation and immune regulation. The nuclear factor kappa B (NF-B) pathway is activated after DCs recognize an inflammatory stimulus. As a result of this activation of the NF-B pathway, MHC class II expression has been found to increase, which primes T cells to be attracted by chemokines released by DCs in synovium or tissue in RA (59). Inflammatory DCs (infDCs), a newly discovered group of DCs, have been identified in RA and have been shown to induce the differentiation of Th17 cells from naïve CD4^+^T cells *via* secretion of IL-23 (60). However, there is evidence that the ability of DCs to engage in cross-presentation and uptake of microbes is reduced in the elderly population due to decreased migration and expression of costimulatory cytokines that are essential for T cell stimulation, which might constitute one of the primary defects in protection against infections in EORA (61, 62). In the temporal arteries of PMR patients, CD83^+^DCs may generate CCL19 and CCL21, and are essential for triggering and attracting T cells (63).

At an early stage of RA, natural killer (NK) cells can be found in synovial tissue; these have been found to trigger the differentiation of osteoclasts. Specifically, NK cells promote the formation of osteoclasts by expressing cell-surface receptor activator of NF-κB ligand (RANKL) and a low level of macrophage colony-stimulating factor (M-CSF) (64). However, it has also been noted that NK cells stimulated by IL-15 might play a protective role by killing osteoclasts through contact-dependent pathways (65). CD56^+^NK cells have been observed in RA and may enhance the ability of CD14^+^ monocytes to secrete TNF (33). Reduced numbers of circulating CD56^dim^ cells have been found in patients with recently diagnosed RA, but this is not the case for the CD56^bright^ subset. This decrease has been found to result from cell apoptosis induced by CD16 signaling following interaction with IgG complexes (66). On the other hand, the enrichment of CD56^bright^ cells in synovial fluid and tissue may be the primary reason for the longstanding inflammatory process in late-stage RA. Moreover, this subset of NK cells in the ordinary age-related population has been found to be shifted from CD56^dim^ to CD56^bright^. Reduced activity is observed in the functions of cytotoxicity and cytokine secretion, as well as migration (67). In the peripheral blood of PMR patients (36), NK cell numbers have been shown to be reduced (44), although the role of these cells in the disease remains unclear.

## Adaptive immune response

T and B cells move into the synovial membrane after being activated by the innate system, leading to persistent inflammation and causing further joint destruction. Multiple subsets of T cells are involved in PMR; however, the role of B cells remains unclear.

### T cells

Various effector subsets divided out from activated CD4^+^T cells play a pivotal role in autoimmune diseases. Th1 cells have been found to be marked by the release of IFN-γ and Th2 cells by IL-4, an anti-inflammation cytokine. Patients with RA have high levels of IFN-γ and low levels of IL-4 in their synovium, indicating that Th1 cells predominate in the disease (68). In contrast, patients with PMR have been shown to have lower levels of Th1 in their peripheral blood (69). However, in synovial fluid and bursal tissue, an increase in Th1 cells rather than Th17 cells and an increase in IFN-γ rather than IL-17 has been observed in PMR (70).

Tregs are a subset of T cells that can modulate the immune response and maintain self-tolerance; an imbalance between Treg and Th17 may result in autoimmune diseases (71). In comparison to healthy controls, patients with RA have a lower incidence of Tregs and higher incidence of Th17 cells in their peripheral blood, which suggests the involvement of an imbalance between Tregs and Th17 at the onset of the disease. In addition, when cultured *in vivo*, effector memory T cells (EMTs) have been found to induce more Th17 cells compared with central T cells and naïve T cells in RA patients, indicating that the amplification of Th17 cells in RA patients may arise through differentiation from EMTs (72). According to a recent study, MaR1 can effectively ameliorate the progression of RA by upregulating mRi21 to correct the Treg/Th17 imbalance (73). The frequency of Treg cells is increased by MTX and biological DMARDs; this alters the Treg/Th17 balance and improves the prognosis of RA patients. Patients with RA can also benefit from JAK inhibitor therapy, although this does not alter the Treg/Th17 balance (74). In patients with PMR, Treg cells are only mildly altered, while levels of Th17 cells are significantly elevated in the peripheral blood and have been found to decrease following glucocorticoid treatment. CD161^+^CD4^+^T lymphocytes, which are thought to be precursors of Th17 cells, are similar in PMR patients and healthy controls, but they may produce more IL-17 *in vitro*. These strands of evidence underline the claim that the pathophysiology of PMR may be influenced by Treg/Th17 imbalance (69).

In patients with RA, natural killer group 2 member D (NKG2D) has been found to be expressed on CD4^+^CD28^−^T cells in peripheral blood and synovial tissue (75). One study has demonstrated a dose-dependent reduction of TNF-α after 24 h when RA synovial membrane cells are cultured with anti-NKG2D monoclonal antibody. Furthermore, clinical scores (with disease severity being assessed by paw thickness) have been found to improve significantly when mice with collagen-induced arthritis receive an intraperitoneal injection of CX5, a monoclonal anti-NKG2D antibody, indicating that anti-NKG2D therapy has a protective effect against joint damage. The authors of this study have also investigated the effects of this type of therapy on NK cells' killing capacity, discovering that anti-NKG2D therapy reduces the amount of IL-17 in CD4^+^ T cells (76, 77). PMR patients exhibit higher expression of NKG2D (which is upregulated by antigenic stimulation and pro-inflammatory cytokines) in circulating CD4^+^CD28^−^T cells and CD8^+^T cells compared with HCs. NKG2D-expressing T cells may also contribute to chronic inflammation in PMR by enhancing the secretion of IFN-γ (32).

Follicular helper T (Tfh) cells, which are effector T cells that help to activate B cells, can be detected in the circulating blood in patients with RA. CD4^+^CXCR5^+^PD-1^hi^T cells have been found to be involved in RA via inducement of B cells to produce more antibodies and an increase in levels of cytokine IL-21. Tph cells have also been found to have the ability to convert B cells into plasma cells. The presence of Tph cells in the synovium in RA suggests that T–B interaction can occur locally in RA. During disease remission, Tfh rather than Tph levels have been found to decrease, indicating the role of the former as a possible marker for RA activity (78, 79). No significant difference has been observed in the alternation of circulating Tfh cells among patients with PMR (80).

Th22 cells, differentiated from CD4^+^T cells induced by IL-6 and TNF-α, are characterized by high levels of secretion of IL-22 (81); they are found in abundance in peripheral blood in RA, and have been found to migrate to synovia *via* CCL28. When cultured with monocytes, Th17, Th1, and Th2 cells do not appear to produce as many osteoclasts as Th22 cells do. Th22 is involved in the bone destruction that occurs in RA via enhancement of the formation of osteoclasts through IL-22 (82). IL-22 has been observed in the inflamed temporal arteries occurring in GCA (83), and may also be involved in the pathogenesis of PMR.

### B cells

In addition to producing antibodies and participating in humoral immune responses, B cells can be transformed into plasma cells, which can act as antigen-presenting cells by processing and presenting antigens. The generation of the autoantibodies RF and ACPA are currently the best-known functions of B cells in RA. Although RA can be diagnosed with greater specificity *via* ACPA, it is still unclear whether APCA contributes to RA by stimulating osteoclastogenesis. ACPA enhances the interaction of CD147 and ITGB1, which in turn activates the PI3K/Akt/NFB pathway, resulting in the synthesis of NOD-like NLRP3 inflammasome and IL-1 (84). Anti-cyclic citrullinated peptide (CCP) titers have been found to be 65% positive in patients with EORA, while they are negative in PMR. The anti-CCP antibody can therefore function as an important marker for the differential diagnosis from EORA to PMR with good sensitivity and specificity, especially for EORA patients with PMR-like symptoms. Similarly, RF has been found to be positive at 66.7% in patients with EORA and 7% in PMR, and RF titers are much higher in EORA than in PMR (423 ± 112 vs. 35.3 ± 2.4, normal range 0–22IU/L). High levels of RF may thus be helpful in distinguishing EORA from PMR (85). Although a substantial correlation has been observed between RF levels and disease activity, and RF positivity is associated with poorer prognosis, the role of RF in the etiology of RA is as yet unknown (86). On the other hand, lower levels of RF in EORA are associated with poorer outcomes, whereas no specific antibodies marking PMR disease activity or diagnosis have yet been discovered.

The function of B lymphocytes has been further investigated since the discovery that anti-CD20 treatment has a positive impact in terms of preventing the progression of RA. Fc-receptor-like-4F (FcRL4) B cells, a subpopulation of memory B cells that express RANKL, have been discovered in synovial tissue in RA. It has also been shown that TNF-α, which can cause bone deterioration, is produced at a significantly higher rate when FcRL4 B cells are present (49). Another subset of B cells, namely CD21^−/*low*^B cells, may also contribute to joint damage by expressing RANKL and proinflammatory cytokines (87). One study has found that dominant BCR clones may be found in the peripheral blood of those at risk of developing RA. In particular, at-risk patients with more than five dominant BCR clones found in their peripheral blood are more likely to develop RA. Once RA has manifested, these BCR clones move to synovial tissue. However, because the at-risk individuals who participated in this study were under 60, it is unclear whether BCR contributes to the development of EORA (88).

Recently, increased expression of CXCL9 and CXCL13 has been observed in the blood of drug-naïve patients with PMR. Baseline CXCL13 expression is correlated with the disease activity index of PMR. Conversely, circulating CXCR3^+^ and CXCR5^+^ switched memory B cells have been found to be decreased, and these are inversely associated with CXCL9 and CXCL13. This study has demonstrated that the CXCL9–CXCR3 and CXCL13–CXCR5 axes play an important role in patients with PMR. Changes in the chemokine and chemokine receptor pathways contribute to the homing and organization of B-cells in the vessel wall (89). B cells are reduced in circulating blood (44) and are rarely found in the synovial fluid in PMR (70). In comparison to controls, patients with PMR have been found to have considerably fewer CD19^+^ B cells and CD24^high^CD38^high^ transitional B cells; the levels of these increase in response to tocilizumab therapy (90).

## Conclusions

**Figure 1:**
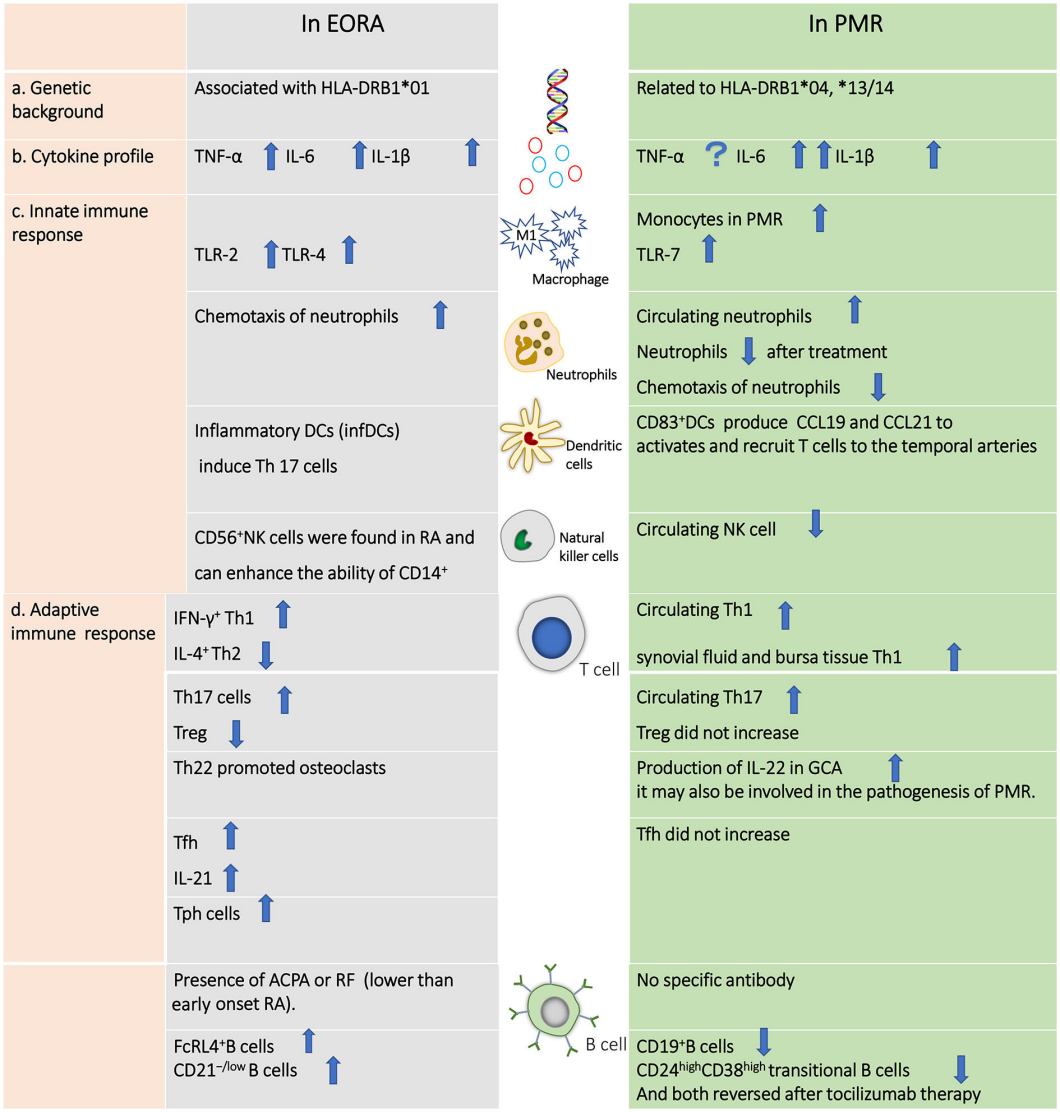
Differences in the pathogenesis of elderly-onset rheumatoid arthritis vs. polymyalgia rheumatic.

There are few published papers involving a head-to-head comparison between EORA and PMR; hence, we have been unable to draw a clear conclusion on the difference in pathogenesis between these two similar diseases (Figure 1). However, EORA and PMR have different genetic backgrounds, in that HLA-DRB1^*^01 is associated with EORA, whereas HLA-DRB1^*^04 and HLA-DRB1^*^13/14 are more closely related to PMR. Given that these two diseases both affect elderly patients, the potential involvement of immunosenescence in their pathophysiology requires further investigation. Predominant macrophages and T cells have been found to be present in the synovial tissue of patients with PMR, while few neutrophils and no B cells, natural killer cells, or gamma/delta T cells are observed in PMR (91). In contrast, NK cells are present in the synovial fluids of RA patients, and a strong correlation with disease severity has been observed (92). Innate immune cells, such as M1 macrophages and neutrophils, are crucial in the development of EORA, and monocytes and neutrophils may function as proinflammatory cells that can trigger the “fire” of PMR. Following innate immune activation, T cells and B cells move to the synovial membrane *via* chemokines, generating a chronic inflammatory response that further damages the joint. Levels of both Th1 and Th17 cells are increased in both of the two diseases. However, Tfh cells are increased in EORA, but not altered in PMR. B cells are highly activated in EORA, with the ability to produce APCA or RF, but B cells are decreased in PMR; the function of B cells is still unknown. TNF-α, IL-6, and IL-1β are all increased in EORA, although IL-6 and IL-1β are also elevated in PMR.

Although the two diseases have similar symptoms, fine distinctions do exist. In PMR, there is a tendency for pain and stiffness to occur in the shoulder and pelvic girdle, while EORA primarily affects the large joints such as the knee, ankle, and sometimes the shoulder and hip joints. ACPA is increased in most patients with EORA, but not in patients with PMR. Therefore, the serum marker ACPA appears to be helpful in making a differential diagnosis of the two diseases. If ACPA is also negative, the presence of synovitis confirmed by ultrasound or MRI scan can confirm a diagnosis of EORA. Extracapsular changes such as bursitis, peritendinitis, capsulitis, or myofascial lesions, confirmed by ultrasound, MRI scan, or PET scan, suggest a diagnosis of PMR.

## Author contributions

WC: conception and design of the study and final approval of the manuscript. JW, FY, XM, and JL: literature review and writing of the manuscript. All authors contributed to the article and approved the submitted version.
